# MYB24 Negatively Regulates the Biosynthesis of Lignin and Capsaicin by Affecting the Expression of Key Genes in the Phenylpropanoid Metabolism Pathway in *Capsicum chinense*

**DOI:** 10.3390/molecules28062644

**Published:** 2023-03-14

**Authors:** Shuang Yu, Wei Zhang, Liping Zhang, Dan Wu, Peixia Sun, Chuang Huang, Genying Fu, Qin Deng, Zhiwei Wang, Shanhan Cheng

**Affiliations:** 1 Sanya Nanfan Research Institute, Hainan University, Sanya 572025, China; yu18285209785@163.com (S.Y.); cocoapuff_93@163.com (D.W.);; 2 College of Horticulture, Hainan University, Haikou 570228, China

**Keywords:** capsaicin, lignin, phenylpropanoid metabolism, pungency regulation, MYB24 transcription factor

## Abstract

The wide application of pepper is mostly related to the content of capsaicin, and phenylpropanoid metabolism and its branch pathways may play an important role in the biosynthesis of capsaicin. The expression level of MYB24, a transcription factor screened from the transcriptome data of the pepper fruit development stage, was closely related to the spicy taste. In this experiment, *CcMYB24* was cloned from Hainan Huangdenglong pepper, a hot aromatic pepper variety popular in the world for processing, and its function was confirmed by tissue expression characteristics, heterologous transformation in *Arabidopsis thaliana*, and VIGS technology. The results showed that the relative expression level of *CcMYB24* was stable in the early stage of pepper fruit development, and increased significantly from 30 to 50 days after flowering. Heterologous expression led to a significant increase in the expression of *CcMYB24* and decrease in lignin content in transgenic *Arabidopsis thaliana* plants. *CcMYB24* silencing led to a significant increase in the expression of phenylpropanoid metabolism pathway genes *PAL*, *4CL*, and *pAMT*; lignin branch *CCR1* and *CAD*; and capsaicin pathway *CS*, *AT3*, and *COMT* genes in the placenta of pepper, with capsaicin content increased by more than 31.72% and lignin content increased by 20.78%. However, the expression of *PAL*, *pAMT*, *AT3*, *COMT*, etc., in the corresponding pericarps did not change significantly. Although *CS*, *CCR1*, and *CAD* increased significantly, the relative expression amount was smaller than that in placental tissue, and the lignin content did not change significantly. As indicated above, *CcMYB24* may negatively regulate the formation of capsaicin and lignin by regulating the expression of genes from phenylpropanoid metabolism and its branch pathways.

## 1. Introduction

Pepper is an annual or perennial plant of the genus Capsicum of Solanaceae. Because of its unique spicy flavor, the pepper is used in food [1], medical treatment [2], industry [3], environmental protection [4,5], military security [6], and other fields. Capsaicin, the main spice ingredient, is mainly synthesized in the placenta tissue of pepper fruit and accumulated in vacuoles of placenta epidermal cells. Its synthesis mainly involves, in phenylpropanoid metabolism, the capsaicin branch pathway (CBP) and branched chain fatty acid metabolism pathway [7,8].

As a ubiquitous and essential secondary metabolic pathway in plants, the phenylpropanoid metabolism pathway leads to the synthesis of downstream branch secondary metabolites such as capsaicin, lignin, flavonoids, and anthocyanins. Phenylalanine was used as the starting substance to form the CoA ester of phenylpropanoid acid under the catalysis of *PAL*, *C4H*, *4CL*, etc., and to form capsaicin, lignin, flavonoids, anthocyanins, and other precursors in different catalytic pathways [9,10]. Capsaicin was synthesized from vanillylamine from the phenylpropanoid metabolic pathway and 8-methyl-6-quillenoic acid CoA formed by the branched-chain fatty acid pathway under *CS* catalysis. In addition to the primary metabolic pathway, it was found that the metabolism of glutamic acid, alanine, lignin, etc., may be related to spicy metabolism [11]. About 50 genes, including the *PAL*, *C4H*, and *4CL* genes involved in phenylpropanoid metabolism, *BCAT*, *KAS*, and *ACS*, participated in the branched-chain fatty acid pathway; essential genes *pAMT* and *AT* for capsaicin biosynthesis, and lignin branch rate-limiting genes *CCR*, *CAD*, etc., were thought to be related to the formation of hot taste [12,13,14]. RNA analysis showed that the expression levels of most genes of the phenylpropanoid metabolism pathway in the pepper placenta were higher than those in other parts. The expression levels of *PAL*, *C4H*, *pAMT*, *AT*, *KAS*, and other genes in high-spicy peppers were much higher than those in low-spicy or non-spicy peppers, and the expression levels peaked at 20 to 40 days after the flowering and pollination of pepper or at the green ripening of pepper fruits [15,16,17]. High-throughput RNA-Seq found that *PAL*, *C4H*, *COMT*, *C3H*, *BCAT*, *FatA*, *ACL*, *pAMT*, and *AT* genes were up-regulated or expressed explicitly in pepper placenta tissues [9]. The silencing and expression levels of *AT3*, *COMT*, *pAMT*, *KAS*, *KR1*, and other genes significantly reduced the synthesis of capsaicin [18,19]. The lignin content in the pericarp and placenta of *CcCCR2* silenced pepper decreased by 18.8% and 22.8%, respectively, while the capsaicin content increased by 16.2% and 19.1%, respectively [20]. It was suggested that differences in the temporal and spatial expression of phenylpropanoid, capsaicin, lignin, and branched chain fatty acid pathway genes might be the reason for the variation of the complex trait capsaicin content.

In plants, the temporal and spatial expression of genes related to quality and resistance is often regulated by transcription factors, especially MYB, which is the most important regulator of phenylpropanoid and branch pathway metabolism. *AtMYB11*, *AtMYB12*, and *AtMYB111* activated the expression of *CHS*, *CHI*, and *F3H*, which promoted the flavonoid biosynthesis, and *AtMYB3*, *AtMYB4*, *AtMYB7*, and *AtMYB32* could inhibit the expression of *PAL*, *C4H*, *4CL*, and *CCR* genes, thereby reducing lignin biosynthesis [21,22]. In the capsaicin synthesis pathway, the expression level of structural genes was correlated with the degree of spiciness and was regulated cooperatively [23,24]. *CaMYB31*, *CaMYB108*, and *CaMYB48* have been proven to be involved in the regulation of capsaicin biosynthesis [25]. *CaMYB48* was found to directly control the expression of acyltransferases and ketoacyl-ACP synthetases and the accumulation of capsaicin-like compounds [26,27]. *ZmMYB111* and *ZmMYB148* in maize activated the activity of *PAL*, thus regulating the synthesis of phenylpropanoid derivatives [28]. In tomatoes, the *SlMYB12* gene was proved to regulate the biosynthesis of flavonol substances [29]. The strawberry *FvMYB24* gene was related to plant salt resistance. Moreover, after overexpression of the *FvMYB24* gene, the expression of significant genes in the SOS pathway, such as AtSOS1, AtSOS2, and AtSOS3, significantly increased [30]. The R2R3-MYB transcription factor *MdMYB24-like* in apples participated in MeJA-induced anthocyanin biosynthesis [31].

Based on the type diversity and complex transcriptional regulation mechanism, more MYB studies are conducive to revealing the regulation mechanism of phenylpropanoid metabolism and the lignin, capsaicin, and other branch pathways. In previous transcriptome sequencing, we found that the expression of *CcMYB24* in pepper fruits showed a similar increasing trend with the change in capsaicin content. In this study, *CcMYB24* was cloned from the Hainan Huangdenglong pepper. It was indicated that it might regulate capsaicin and lignin biosynthesis by affecting phenylpropanoid metabolism and the expression of branch genes.

## 2. Results

### 2.1. Cloning and Sequence Analysis of CcMYB24 Transcription Factors

**Figure 1:**
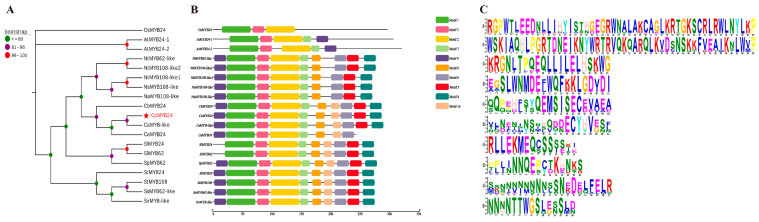
Analysis of *CcMYB24* gene structure and conserved motifs. (**A**) *CcMYB24* protein phylogenetic tree; (**B**) conservative motif map; and (**C**) gene structure. Note: OsMYB24 (XP_015624260.1), AtMYB24-1 (AT5G49620.1), AtMYB24-2 (AT5G49620.2), NtMYB62-like (XM_009627940.3), NtMYB108-like2 (XM_016613012.1), NtMYB108-like1 (XM_016595182.1), NsMYB108-like (XM_009799649.1), NaMYB108-like (XM_019401273.1), CbMYB24 (CQW23_25186_mrna), CcMYB24 (BC332_27190_mrna), CaMYB-like (XM_016691132.2), CaMYB24-like (PHT67336), SlMYB24 (rna-XM_004239365.4), SlMYB62 (XM_004239365.4), SpMYB62 (XM_015220269.2), StMYB24 (rna-XM_006344239.2), StMYB108 (XM_006344239.2), SsMYB62-like (XM_049547997.1), SvMYB-like (XM_049500874.1).

The sequenced results showed that the full length ORF of the *CcMYB24* r gene was 864 bp, encoding 287 aa. By analyzing the physicochemical properties of *CcMYB24* protein, we found that it has the following essential characteristics: the molecular formula was C_2666_H_4470_N_864_O_1100_S_126_; the molecular weight was 70,267.51 Da; the theoretical isoelectric point pI was 5.18; it does not contain a signal peptide and was a non-secretory protein; there was no transmembrane structure; and it was theoretically localized in the nucleus, indicating that this transcription factor may regulate gene transcription. In addition, the conserved structural domain was viewed using NCBI CDD and was found to be a typical R2R3-MYB structural domain. The evolutionary analysis revealed that the homologs of *CcMYB24* were mainly CaMYB-like and CaMYB24 in *Capsicum annuum* and CbMYB24 in *Capsicum baccatum* (Figure 1A). Conserved motifs and gene structure maps of the MEME predicted analysis proteins are shown in Figure 1B,C.

### 2.2. Differential Expression of CcMYB24 in Different Growth and Development Stages of Pepper

**Figure 2:**
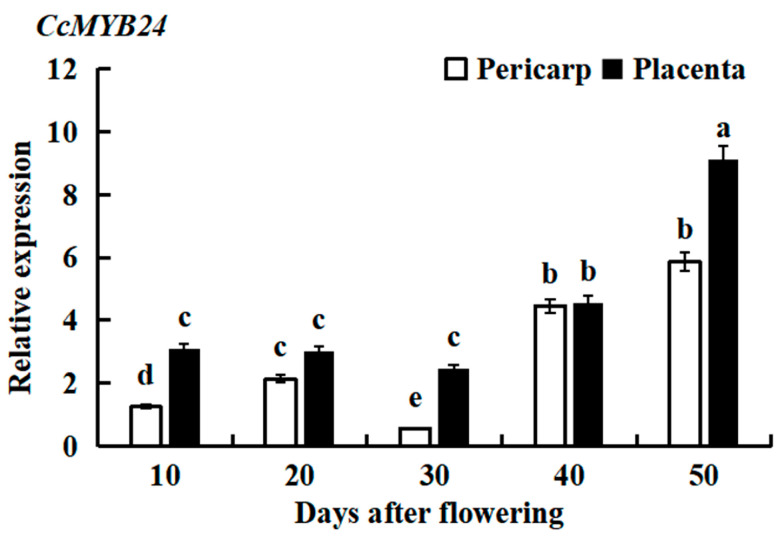
The relative expression levels of *CcMYB24* in pepper at different tissues and growth stages. Each value represents the mean ± standard of three replicates. a, b, c, d, and e indicate significant differences in values at *p* < 0.05.

The qRT-PCR analysis of Huangdenglong pepper fruit showed that the expression level of *CcMYB24* was generally stable 10 to 30 days after flowering (Figure 2), but increased rapidly after 30 days. Compared with 30 days, the relative expression increased by about four times at 50 days after flowering. The relative expression level of *CcMYB24* in placenta tissue was significantly higher than that in the pericarp. Because capsaicin is mostly synthesized in placenta tissue, it is believed that this difference in expression is related to the formation of spicy taste.

### 2.3. Heterologous Expression of the CcMYB24 Gene in A. thaliana

**Figure 3:**
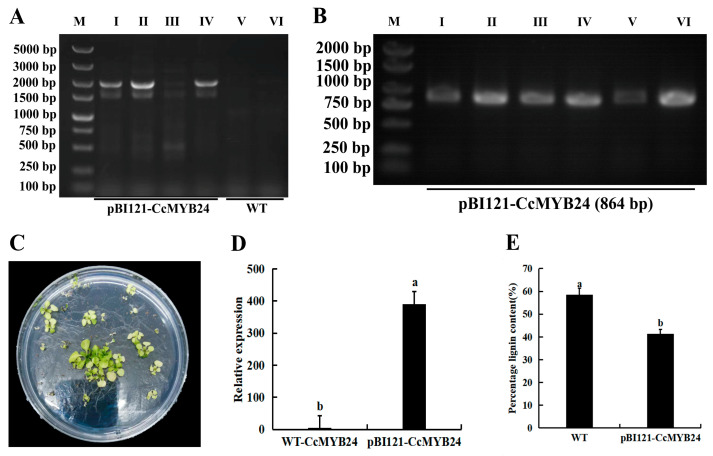
T_2_-generation of pBI121-*CcMYB24* transgenic *A. thaliana* detection. (**A**) T_2_-generation transgenic *A. thaliana* PCR detection, M: DL5000 marker, I–IV: *CcMYB24 A. thaliana* overexpression lines, V–VI: wild-type *A. thaliana* plants; (**B**) T_2_-generation transgenic *A. thaliana* RT-PCR detection, M: DL2000 marker, I–VI: *CcMYB24 A. thaliana* transgenic lines; (**C**) T_2_-generation resistance screening; (**D**) relative expression in T_2_-generation transgenic *A. thaliana*; and (**E**) percentage of lignin content in transgenic *A. thaliana*. Each value represents the mean ± standard of three replicates. a and b indicate significant differences in values at *p* < 0.05. Note: pBI121-*CcMYB24*: *CcMYB24 A. thaliana* overexpression lines; WT: wild-type *A. thaliana* plants.

The seeds of infected *A. thaliana* were harvested and then transformed into the plates to screen the T_2_-generation positive seedlings (Figure 3C). Then, the RNA of T_2_-generation transgenic plants were extracted, and the cDNA was synthesized by reverse transcription PCR. Using pB-*CcMYB24*-R/F as primers, the actual length of the amplified fragment was about 2044 bp (since the M13 site was located on both sides of the insertion point of the target segment, the actual band length was 1180 bp + 864 bp) in transgenic *A. thaliana* plants, while the target band was not amplified from wild-type *A. thaliana* (Figure 3A). At the same time, the PCR reaction was performed using *A. thaliana* cDNA as a template, and the expected bands could be observed by gel electrophoresis (Figure 3B), which indicates that the *CcMYB24* gene has been transferred into the *A. thaliana* genome and transcription has occurred, allowing the next step of the experiment. The relative expression of *CcMYB24* in wild-type and transgenic *A. thaliana* showed that *CcMYB24* had been overexpressed in transgenic plants (Figure 3D). Lignin content determination of the stems of T_2_-generation transgenic *A. thaliana* and wild-type *A. thaliana* plants showed that the lignin content was reduced by 29.47% in *CcMYB24* transgenic *A. thaliana* compared with wild-type *A. thaliana* (Figure 3E).

It is speculated that the *CcMYB24* inhibits phenylpropanoid lignin branching metabolism after being transferred into *A. thaliana*, so *CcMYB24* may also play an inhibitory role in the lignin synthesis stage of pepper.

### 2.4. VIGS Identification of CcMYB24 in Hainan Huangdenglong Pepper

**Figure 4:**
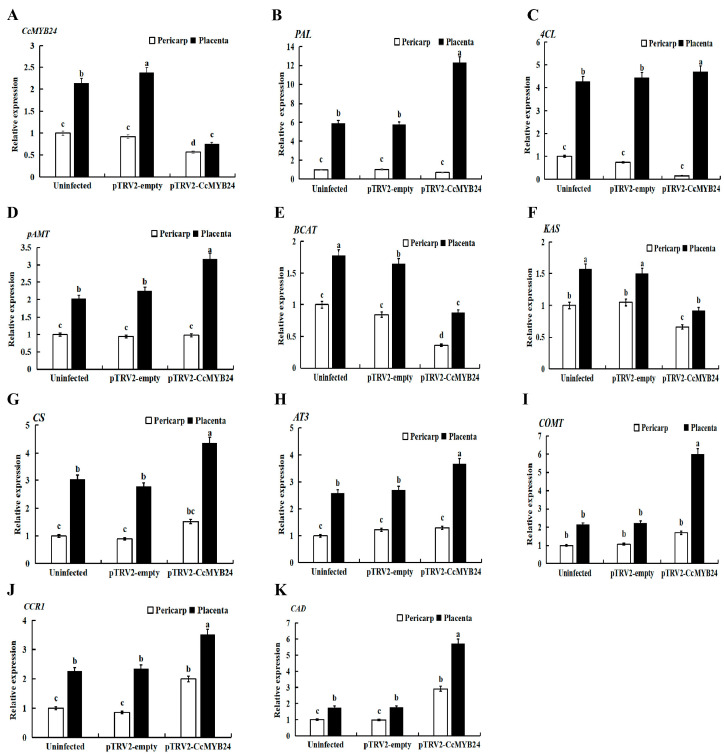
Expression changes of genes related to capsaicin biosynthesis after *CcMYB24* silencing. (**A**) Changes of *CcMYB24* gene expression after gene silencing and (**B**–**K**) changes in the expression of major genes in capsaicin biosynthesis pathway after gene silencing. Each value represents the mean ± standard of three replicates. a, b, c, and d indicate significant differences in values at *p* < 0.05.

The analysis of qRT-PCR showed that the expression level of *CcMYB24* decreased by 43.62% and 64.71% in the pericarp and placenta of infected pepper compared with the uninfected plants, respectively (Figure 4A). Further detection showed that the relative expression of *PAL*, *4CL*, *pAMT*, *BCAT*, and *KAS* genes in the pericarp of the silenced plant fruit decreased by 28.73%, 83.87%, 2.01%, 63.93%, and 33.86%, respectively, while the relative expression of *CS*, *AT3*, *COMT*, *CCR1*, and *CAD* in the pericarp increased by 52.19%, 29.75%, 69.81%, 99.20%, and 191.49%, respectively, the relative expression of *BCAT* and *KAS* genes in the fruit placenta increased by 50.67% and 41.17%, respectively, and the relative expression of *PAL*, *4CL*, *pAMT*, *CS*, *AT3*, *COMT*, *CCR1*, and *CAD* increased by 108.20%, 10.03%, 56.69%, 43.48%, 42.54%, 180.53%, 55.46%, and 222.80%, respectively (Figure 4B–K).

**Figure 5:**
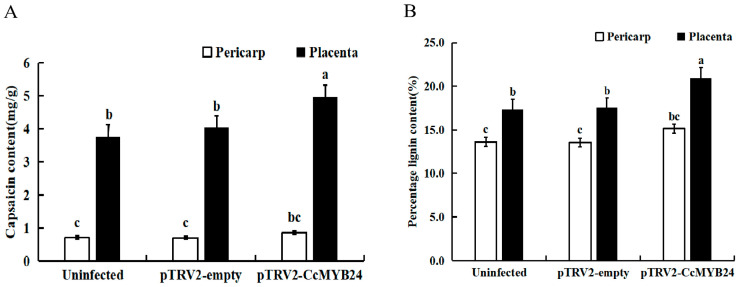
Changes of capsaicin and lignin contents after gene silencing treatment. (**A**) Capsaicin content and (**B**) lignin content. Each value represents the mean ± standard of three replicates. a b and c indicate significant differences in values at *p* < 0.05.

The analysis of capsaicin content in pepper with different treatments showed that the content increased by 7.22% in the mature fruit placenta tissue of plants infected with blank vectors, while it decreased by 8% in the pericarp. The capsaicin content of plants infected with blank vectors was not significantly different from that of untreated plants (Figure 5A). The changes of capsaicin in the pericarp and placenta of pepper after gene silencing treatment were different. The capsaicin content in the pericarp of pTRV2-*CcMYB24* silenced plants increased by 21.33% compared with the untreated group, and increased by 31.72% in the placenta, respectively. There was no significant difference in lignin content in the placenta tissue and pericarp of fruit between the non-infected and blank vector-infected pepper, with an increase of 0.97% in the placenta and a decrease of 0.55% in the pericarp (Figure 5B). However, in the plants silenced by pTRV2-*CcMYB24*, the lignin content in the pericarp and placenta tissue increased by 11.16% and 20.78%, respectively, indicating that *CcMYB24* had a significant effect on the lignin content in the placenta tissue.

Compared with the plants without silencing, the expression of *PAL*, *4CL*, and *pAMT*-related genes in the phenylpropanoid metabolism pathway in plants infected with pTRV2-*CcMYB24* decreased in the pericarp and increased in the placenta. In the fruits and placentas of plants infected with pTRV2-*CcMYB24*, the expression of *BCAT* and *KAS* genes related to the branched-chain fatty acid pathway decreased; the expression of *CCR1* and *CAD* genes related to the lignin pathway and *CS*, *AT3*, and *COMT* genes related to the capsaicin pathway increased; the capsaicin content increased; and the lignin content increased. It was speculated that *CcMYB24* has an inhibitory effect on both capsaicin and lignin synthesis.

## 3. Discussion

Capsaicin biosynthesis is a branch of phenylpropanoid metabolism in pepper. Its synthesis is regulated by a series of structural genes and transcription factors, and MYB transcription factors are an important regulatory gene in plants [32]. In peppers, only the MYB [33,34], WRKY [35], and ERF [36] transcription factor families were found to be associated with the regulation of spiciness. MYBs are widely involved in the regulation of phenylpropanoid metabolism [22,37,38,39,40] and gene expression in various branch pathways, such as the lignin [39], flavonoids [40], anthocyanidins [41,42], and proanthocyanidins [43] pathways. They are also involved in morphological formation regulation, stress, and other physiological activities.

Among plant MYBs, the most common are R2R3-MYB transcription factors, which regulate various biological processes such as tissue development, the abiotic stress response, and metabolism. Many genes and enzymes involved in capsaicin biosynthesis have been identified, cloned, and studied [44]. However, there are only a few studies on the MYB transcription factor of *Capsicum chinense*, and this study cloned the *CcMYB24* transcription factor from the Hainan Huangdenglong pepper to view its conserved domain, which is the typical R2R3-MYB. It was subcellular in the nucleus through bioinformatics analysis, suggesting that it has a regulatory role. However, the research group speculated that the *CcMYB24* gene was negatively correlated with the content of capsaicin, and *CcMYB24* may affect the accumulation of capsaicin by regulating the synthesis of flavonoids, anthocyanins, and lignin. Hence, the heterologous expression of the *CcMYB24* gene confirmed its function in *A. thaliana* and VIGS in pepper. Heterologous expression is often performed on the model plant *A. thaliana* to speculate on the function of plant genes [45,46].

In this experiment, the expression of genes related to phenylpropanoid metabolism in pTRV2-free plants did not change much compared with plants without gene silencing, consistent with previous studies [19]. Studies have shown that genes such as *pAMT*, *BCAT*, *Ca4H*, *KAS*, *PAL*, *4CL*, *CS*, and *AT3* were positively correlated with capsaicin synthesis [19,47,48,49]. Compared with the plants without silencing, the expression of *PAL*, *4CL*, and *pAMT*-related genes in the phenylpropanoid metabolism pathway in plants infected with pTRV2-*CcMYB24* decreased in the pericarp, and it increased in the placenta, which may be related to the synthesis of capsaicin being mainly in the placenta. In the fruit and placenta of plants infected with pTRV2-*CcMYB24*, the expression of related genes *BCAT* and *KAS* in the branched-chain fatty acid pathway decreased; the expression of related genes *CCR1* and *CAD* in the lignin pathway and *CS*, *AT3*, and *COMT* in the capsaicin pathway increased; the capsaicin content increased, and the lignin content increased. It is speculated that *CcMYB24* has an inhibitory effect on both capsaicin synthesis and lignin synthesis. The effect of *CcMYB24* may be to activate the synthesis of related substances in other metabolic branches of the phenylpropanoid pathway, and the specific function needs to be further tested for identification.

## 4. Materials and Methods

### 4.1. Experimental Materials

Hainan Huangdenglong pepper (*Capsicum chinense* Jacq.) was from the pepper research group of the College of Horticulture, Hainan University. The full seeds were selected and soaked in a 55 °C thermostat water bath for 20 min, soaked in 0.1% potassium permanganate for 15 min, rinsed under running water for 2 min, and soaked in room temperature water for 12 h. Then, the seeds were placed in a Petri dish covered with paper towels, poured with water, exposed to 28 °C light for 16 h and 22 °C dark for 8 h to promote germination, and then seeded in a hole dish; after being exposed to white light, they grew to about six true leaves and were transplanted in pots.

Wild-type Arabidopsis thaliana (Columbia) was granted by the College of Tropical Crops, Hainan University. The seeds were placed in the refrigerated layer at low temperature and vernalized for 3 days, The seeds were evenly sprinkled on the nutrient soil and moisturized with plastic wrap. After the cotyledon was unfolded, the seeds were transplanted, and the seeds were exposed to light at 23 °C for 16 h and dark at 18 °C for 8 h, and the side branches grew more unopened buds in about four weeks for transformation.

### 4.2. Main Reagents

The FastPure^®^ Plant Total RNA Isolation Kit (RC401), HiScript^®^III 1st Strand cDNA Synthesis Kit (R312), HiScript^®^III All-in-one RT SuperMix Perfect for qPCR (R333), 2 × Phanta^®^ Max Master Mix (P515), 5min^TM^ TA/Blunt-Zero Cloning Kit (C601), ChamQ Universal SYBR qPCR Master Mix (Q711), DH5α Competent Cell (C502), FastPure Plasmid Mini Kit (DC201), and ClonExpress^®^ Ultra One Step Cloning Kit (C115) all refer to the instruction manual of Vazyme Biotech Co., Ltd. (Nanjing, China). A Gel Extraction Kit (CW2302M) was purchased from Cowin Biotech Co., Ltd. (Beijing, China) GV3101 Chemically Competent Cell (AC1001) was used according to the manufacturer’s protocol of Shanghai Weidi Biotechnology Co., Ltd. (Shanghai, China). Restriction enzymes from New England Biolabs (Beijing, China) LTD. were used. The primers were synthesized by Sango Biotech (Shanghai, China) Co., Ltd. Lignin test kits all refer to the instruction manual of Beijing Solarbio Science & Technology Co., Ltd. (Beijing, China). Capsaicin test kits were purchased from Beacon. Guangzhou Tianyihuiyuan Co., Ltd. (Guangzhou, China), completed sequencing. The remaining reagents were domestically produced for analytical purity.

### 4.3. Extraction of Total RNA and Synthesis of the First Strand of cDNA

The total RNA from pepper young leaves was extracted using a FastPure^®^ Plant Total RNA Isolation Kit (RC401), and the concentration and purity of the extracted RNA were determined by Nanodrop ONE and agarose gel electrophoresis, and stored at −20 °C for later use. The first strand of cDNA was synthesized by reference to the HiScript^®^III 1st Strand cDNA Synthesis Kit (R312).

### 4.4. Cloning of the CcMYB24 Transcription Factor

According to the protein annotation information in the genome of *Capsicum chinense* on NCBI, *CcMYB24* (PHU01939.1), primer premier 5.0 was used to design the cloning primers *CcMYB24*-F/R (Appendix A), using cDNA as the template for PCR amplification, 2 × Phanta Max Master Mix 25 μL, *CcMYB24*-F 2 μL, *CcMYB24*-R 2 μL, cDNA 5 μL, and ddH_2_O 16 μL. There was predenaturation at 95 °C for 3 min, denaturation at 95 °C for 15 s, annealing at 52 °C for 15 s, extension at 72 °C for 30 s, 35 extension cycles, 72 °C, and final extension for 5 min, 4 °C storage. The amplification products were detected by 1% agarose gel electrophoresis. The fragments of interest were purified and recovered by Cowin Biotech Century’s DNA Gel Extraction Kit, connected to the pCE2-TA vector by 5min^TM^ TA/Blunt-Zero Cloning Mix, and then transformed into DH5α Competent Cell. The bacterial solution PCR was verified correctly and sent to Guangzhou Tianyihuiyuan Co., Ltd. for sequencing.

### 4.5. Bioinformatics Analysis of the CcMYB24 Gene

The nucleotide and amino acid sequences were compared by DNAMAN software. The Expasy-ProtParam tool (http://web.expasy.org/protparam/ (accessed on 18 November 2022) was used to predict its physicochemical properties; SignalP-5.0 Server (https://services.healthtech.dtu.dk/service.php?SignalP-5.0 (accessed on 18 November 2022)) to predict its protein signal peptide; TMHMM 2.0 (https://services.healthtech.dtu.dk/service.php?TMHMM-2.0 (accessed on 18 November 2022) to predict the protein transmembrane structure; PSORT II Prediction (https://psort.hgc.jp/form2.html (accessed on 18 November 2022) to predict subcellular localization; and CDD (http://www.ncbi.nlm.nih.gov/Structure/cdd/wrpsb.cgi (accessed on 18 November 2022) to examine its conservative domains. MEME (http://meme-suit.org/index.html (accessed on 28 December 2022) was used to predict conserved motifs of proteins, and MEGA5.0 software and Evoview (https://evolgenius.info//evolview-v2 (accessed on 28 December 2022) were used to map the MYB24 protein sequence evolutionary tree of *CcMYB24* and other species.

### 4.6. qRT-PCR Analysis of CcMYB24 in Different Growth and Development Stages of Pepper

RNA was extracted from Hainan Huangdenglong pepper pericarp and placenta at 10, 20, 30, 40, and 50 days after flowering, respectively, and was reversely transcribed into cDNA. Specific primers *QMYB24*-F/R and *Actin*-F/R (Appendix A) were designed using Primer Premier 5.0 based on *CcMYB24* and Actin (AY486137.1) mRNA sequences. *CcMYB24* was detected using a real-time PCR machine (Applied Biosystems by Thermo Fisher Scientific, Waltham, MA, USA) for *CcMYB24* at different stages of growth and development. The qRT-PCR reaction system was: 2 × ChanQ Universal SYBR qPCR Master Mix, 10 μL; *QMYB24*-F and *QMYB24*-F, 0.4 μL each; cDNA, 2 μL; and ddH2O, 7.2 μL. The qRT-PCR reaction procedure was: 50 °C for 3 min, 95 °C for 30 s, 95 °C for 5 s, 60 °C for 30 s (fluorescence acquisition), and 72 °C for 30 s (fluorescence acquisition) for 40 cycles. The dissolution curve program was: 95 °C for 25 s, 60 °C for 60 s, and 95 °C for 1 s.

### 4.7. Heterologous Expression of CcMYB24 in A. thaliana

Based on cloning *CcMYB24* in Hainan Huangdenglong pepper, *ScaI* and *XBaI* digestion sites on the pBI121 vector were selected and primers pB-*CcMYB24*-F/R with homologous arms were designed (Appendix A). The plasmid extracted from the sequenced *CcMYB24* gene solution was used as a template to obtain fragments with homologous arms and digestion sites. The pBI121 no-load plasmid was linearized by endoenzyme digestion of *ScaI* and *XBaI*, followed by gel recovery. The insert with the homologous arm and digestion site was attached to the linearization vector. The homologous recombinant plasmid was converted into DH5α, single colonies were singled for PCR and sequencing verification, and the plasmid pBI121-*CcMYB24* plant heterologous expression vector was extracted from the sequenced homologous recombinant plasmid to GV3101 Chemically Competent Cell.

The *CcMYB24* gene was transformed with reference to *A. thaliana* inflorescence infection [50,51,52]. The total DNA of transgenic *A. thaliana* was extracted by the CTAB method. Positive plants identified correctly by PCR were continued in culture to collect T_2_ seeds. T_2_-generation seeds were screened for resistance using 50 μg/mL Kan. The total RNA of T_2_-generation *A. thaliana* leaves was extracted, and the expression of the *CcMYB24* gene in transgenic *A. thaliana* plants was detected by qRT-PCR; *A. thaliana* plants with high expression were selected. Wild-type *A. thaliana* plants up to eight weeks old and T_2_-generation transgenic *A. thaliana* plants with positive screening verification and high expression were dried at 80 °C, ground, and subjected to a 40-mesh sieve, and 5 mg was weighed for lignin assay.

### 4.8. VIGS of CcMYB24 in Hainan Huangdenglong Pepper

*CcMYB24*-specific fragments of about 400 bp were selected, the digestion sites *BamHI* and *EcoRI* were added at the 5′ end of the upstream and downstream primers, and a homologous arm of the pTRV2 vector of about 20 bp was added, respectively, and the primer pT-*CcMYB24*-F/R with a homologous arm (Appendix A) was designed. The homologous recombination method of the pTRV2-*CcMYB24* plant heterologous expression vector was obtained.

When the fruits reached the green ripening stage, the plants with the same growth were divided into three experimental groups; each experimental group had three peppers: one group was pTRV2 unloaded, one group was pTRV2-*CcMYB24*, and another group was a blank control. Except for the blank control group, all other groups needed to be mixed with pTRV1 without load and injected. An amount of 100 μL of pTRV2 no-loaded Agrobacterium, pTRV2-*CcMYB24* Agrobacterium, and pTRV1 Agrobacterium was added to 10 mL of YEB liquid medium (25 μg/mL Kan, Rif and Gen) at 28 °C, 200 rpm, and cultured for 16–20 h to make the OD_600_ value between 1.0 and 1.2. Then, to 1 mL of the cultured bacteria, 25 mL of IM induction medium was added at a ratio of 1:25 (NaH_2_PO_4_, glucose and MES) at 28 °C, 200 rpm, and cultured for 16 h, so that the OD_600_ value was about 0.9. Then, the cultured bacteria were centrifuged at 4 °C, 3500 rpm for 10 min to collect the bacteria, 25 mL of MES was added to resuspend the bacteria, and they were centrifuged again at 4 °C for 10 min. Next, the bacteria were resuspended with 1/2 volume of MES to an OD_600_ of about 1.0, and 50 μL of 200 mM AS was added per 25 mL pTRV1 (the final concentration of AS was 400 μM, and the bacteria OD_600_ was about 2.0). pTRV1 was mixed with pTRV2 no-load and pTRV2-*CcMYB24* at 1:1, respectively, so that the final concentration of AS was 200 μM, with an OD_600_ of about 1.0, and was placed in the dark at about 23 °C for 3 h. In the placenta of the young fruit, about five days after injection of pepper flowers, the plants were dark treated at 16 °C for 24 h, then placed in light at 24 °C/16 h and dark at 22 °C/8 h to continue incubation. After the pepper was ripe, the content of capsaicin and lignin in the pericarp and placenta of the pepper, as well as the expression of genes related to phenylpropanoid metabolism, were measured. The genes and primers determined are shown in Appendix A. Actin was the internal reference, and the qRT-PCR reaction system and procedure were consistent with 4.6.

### 4.9. Statistical Analysis

All experiments were repeated three times. Data are presented as the mean ± standard error. Statistical comparisons of the data obtained were performed by SPSS. The data were statistically analyzed by using Student’s *t*-test, with *p* < 0.05 being considered significant.

## 5. Conclusions

In this study, we analyzed the expression characteristics of the *CcMYB24* gene, which was hypothesized to be negatively correlated with capsaicin content by the group in the previous stage. We confirmed its function by heterologous expression of the *CcMYB24* gene in *A. thaliana* and VIGS in pepper. The results indicate that *CcMYB24* may play an inhibitory role in the lignin synthesis pathway in *A. thaliana*. It is determined that *CcMYB24* can inhibit the synthesis of capsaicin and lignin, but whether it also affects the expression of other branch-related genes of phenylpropanoid metabolism and the synthesis of metabolites remains to be further studied.

## Data Availability

Not applicable.

## Sample Availability

Samples of the compounds are available from the authors.

## Appendix A. Primer Sequences

**Table A1:** The lowercase portion of the primer sequence above is the homology arm.

Primer	Sequence (5′–3′)	Use
CcMYB24-F	ATGAGTAGTAATAATAATAATAATAATTTATCATC	Clone
CcMYB24-R	TCAAATATCTACATCTCCTAGC	Clone
pB-CcMYB24-F	gagaacacgggggactctagaATGAGTAGTAATAATAATAATAATAATTTATCATC	Overexpression
pB-CcMYB24-R	cgatcggggaaattcgagctcTCAAATATCTACATCTCCTAGC	Overexpression
pT-CcMYB24-F	gtgagtaaggttaccgaattcGAAGCAATAAAGAATCTATGGGTA	VIGS
pT-CcMYB24-R	cgtgagctcggtaccggatccACAATGATCCTTCATTAGTAAACC	VIGS
QMYB24-F	AATAGCAAGAAGTTTGTTGAAGCA	qRT-PCR
QMYB24-R	TAAGGGTAAATTTGGAAATGAAAG	qRT-PCR
Actin-F	GTCCATCTGCTCTCTGTTG	qRT-PCR
Actin-R	CACCCCAAGCACAATAAGAC	qRT-PCR
PAL-F	TGTCCCGTTGTCCTACATTGCT	qRT-PCR
PAL-R	CTCGGGCTTTCCATTCATCAC	qRT-PCR
4CL-F	CTTCTTCTCAACCATCCCAACA	qRT-PCR
4CL-R	ACGAAATCCTTGACTTCATCCTC	qRT-PCR
pAMT-F	TGGATTTGGAAGACTTGGGACA	qRT-PCR
pAMT-R	GCTTACAAGGACAGCGGCA	qRT-PCR
COMT-F	TAGCACATAACCCAGGAGGCA	qRT-PCR
COMT-R	CACAGCACACCTTACG GAATCT	qRT-PCR
KAS-F	TCGGCTATTGGTGTTGGTGGT	qRT-PCR
KAS-R	CACCTCCCAAGTATTCCGCTA	qRT-PCR
AT3-F	TCAAATGGCAGTTTCCCTTCTC	qRT-PCR
AT3-R	TCAAATGGCAGTTTCCCTTCTC	qRT-PCR
CAD-F	TATGGCACCAGAACAAGCAG	qRT-PCR
CAD-R	CTCCAAGTCCCAATATTCCAC	qRT-PCR
CCR1-F	GATCCAGAACAAATGGTGGAGC	qRT-PCR
CCR1-R	CCAGCAAGTCTCGTCCA CAAC	qRT-PCR
CS-F	TTGGCTCGCGTATAATGACTT	qRT-PCR
CS-R	TGCCGCTGGAATAACACCTC	qRT-PCR
